# Combined Programmed Intermittent Bolus Infusion With Continuous Infusion for the Thoracic Paravertebral Block in Patients Undergoing Thoracoscopic Surgery: A Prospective, Randomized, and Double-blinded Study

**DOI:** 10.1097/AJP.0000000000001037

**Published:** 2022-04-20

**Authors:** Lin Yang, Xinyi Huang, Yulong Cui, Yangfan Xiao, Xu Zhao, Junmei Xu

**Affiliations:** * Department of Anesthesiology, The Second Xiangya Hospital, Central South University; † Hunan Province Center for Clinical Anesthesia and Anesthesiology, Research Institute of Central South University, Changsha, Hunan Province; ‡ Department of Anesthesiology, The First Affiliated Hospital, Sun Yat-sen University, Guangzhou, Guangdong Province, China

**Keywords:** paravertebral block, programmed intermittent bolus infusion, continuous infusion, ropivacaine, thoracoscopic surgery

## Abstract

**Background:**

Continuous thoracic paravertebral block (TPVB) connected with patient-controlled analgesia (PCA) pump is an effective modality to reduce postoperative pain following thoracic surgery. For the PCA settings, the programmed intermittent bolus infusion (PIBI) and continuous infusion (CI) are commonly practiced. However, the comparative effectiveness between the 2 approaches has been inconsistent. Thus, the aim of this study was to explore the optimal PCA settings to treat postthoracotomy pain by combing PIBI and CI together.

**Methods:**

All enrolled patients undergoing thoracoscopic surgery accepted ultrasound-guided TPVB catheterization before the surgery and then were randomly allocated in to 3 groups depending on different settings of the PCA pump connecting to the TPVB catheter: the PIBI+CI, PIBI, and CI groups. Numerical Rating Scales were evaluated for each patient at T1 (1 h after extubation), T2 (12 h after the surgery), T3 (24 h after the surgery), T4 (36 h after the surgery), and T5 (48 h after the surgery). Besides, the consumptions of PCA ropivacaine, the number of blocked dermatomes at T3, and the requirement for extra dezocine for pain relief among the 3 groups were also compared.

**Results:**

First, the Numerical Rating Scale scores in the PIBI+CI group were lower than the CI group at T2 and T3 (*P*<0.05) when patients were at rest and were also lower than the CI group at T2, T3, and T4 (*P*<0.01) and the PIBI group at T3 when patients were coughing (*P*<0.01). Second, the 2-day cumulative dosage of PCA in the PIBI+CI group was lower than both the CI and PIBI groups (*P*<0.01). Third, the number of blocked dermatomes in the PIBI and PIBI+CI groups were comparable and were both wider than the CI group at T3 (*P*<0.01). Finally, a smaller proportion (not statistically significant) of patients in the PIBI+CI group (5.26%, 2/38) had required dezocine for pain relief when compared with the PIBI group (19.44%, 7/36) and the CI group (15.79%, 6/38).

**Conclusions:**

The combination of PIBI and CI provides superior analgesic modality to either PIBI or CI alone in patients undergoing thoracoscopic surgery. Therefore, it should be advocated to improve the management of postoperative pain, clinical outcomes, and ultimately patient satisfaction.

Postthoracotomy pain is regarded as one of the fiercest pain during perioperative periods.^1^ Although the minimally invasive thoracoscopic technique has been widely practiced and markedly reduced pain degree compared with traditional open-chest surgery,^2^ moderate to severe pain still takes place and proves to be associated with impaired lung secretion clearance, delayed mobilization, and delayed ventilatory recovery after surgery,^3^,^4^ leading to a higher rate of postoperative pulmonary complications. Thus, pain management after thoracic surgery remains challenging.

The thoracic paravertebral block (TPVB) now has been recognized as an effective way to reduce postthoracotomy pain.^5^,^6^ Substantial evidence has demonstrated that continuous TPVB through ultrasound-guided catheterization into the paravertebral space provides equivalent pain relief to the epidural analgesia^7^–^9^ and with fewer side effects (eg, hypotension, pulmonary muscle weakness, and urinary retention).^10^,^11^ To date, 2 approaches setting for the patient-controlled analgesia (PCA) pump connecting to the TPVB catheter, including the programmed intermittent bolus infusion (PIBI) and continuous infusion with constant speed (CI) have been widely investigated.^12^–^17^ Between them, PIBI has proven to provide a more rapide and wider dermatomal spread of sensory block than CI.^12^,^13^ Nevertheless, rapid infusion during the PIBI approach may cause increased absorption of local anesthetics into the bloodstream, which may lead to some side effects, including local anesthetic poisoning.^12^,^13^ Meanwhile, results regarding the efficacy of pain control for PIBI and CI are inconsistent. A growing body of evidence has shown that the PIBI provides superior postoperative pain relief to CI.^14^ While other studies have found that PIBI only provides comparable,^15^,^16^ or even inferior analgesia to CI.^17^ Thus, the optimal approach for the TPVB to control the postoperative pain still remains debatable.

In light of the evidence above, we hypothesized that the combination of PIBI and CI could take advantages of both approaches and provide better and safer postoperative analgesia. We conducted a randomized trial in which patients were assigned to receive either PIBI+CI, PIBI, or CI to find an optimal PCA settings to treat postthoracotomy pain.

## METHODS

### Trial Design

This single-center, 3-arm, prospective randomized trial was conducted at the Second Xiangya Hospital of Central South University, in accordance with the principles of the Declaration of Helsinki. The trial was approved by the local ethics committee (NO. 2019.164) and prospectively registered in the Chinese Clinical Trial Registry (www.chictr.org.cn) with the registration number of ChiCTR1900025277. Written informed consents were obtained from all patients participating in the trial.

### Participants

Adult patients with an American Society of Anesthesiologists Physical Status class of I-II undergoing elective unilateral thoracoscopic wedge resection were eligible for this study. The exclusion criteria included: (1) patients with cardiac, hepatic, or renal diseases; (2) patients with inflammation at the TPVB insertion site; (3) patients with a history of allergy to local anesthetics; (4) patients with a history of chronic pain treated with opioids; and (5) pregnant patients.

### Randomization and Blinding

After signing the informed consent, patients were randomly assigned to 3 intervention groups (ie, PIBI+CI, PIBI, and CI) using computer-generated random numbers contained in sealed envelopes. The patients, anesthesiologists, surgeons, and nursing staff were all blinded to the group allocation.

### TPVB and Catheterization

Patients were monitored with a 5-lead electrocardiograph, invasive arterial pressure, and pulse oximetry after entering the operating room. Then, patients received 1 mg of midazolam and 5 μg of sufentanil intravenously before starting the intervention. All TPVB and catheterization in this study were performed by the same anesthesiologist (J.X.), who was blinded to the group allocation.

**FIGURE 1:**
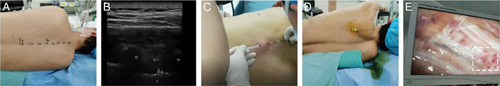
The thoracic paravertebral block procedure. A, Anatomic location of the thoracic vertebrae. B, Ultrasound-guided exposure of the paravertebral space. C, Fixation of the catheter by building an intradermal tunnel. D, Completion of the thoracic paravertebral block. E, The location of the paravertebral block catheter viewed through the thoracoscopy.

**FIGURE 2:**
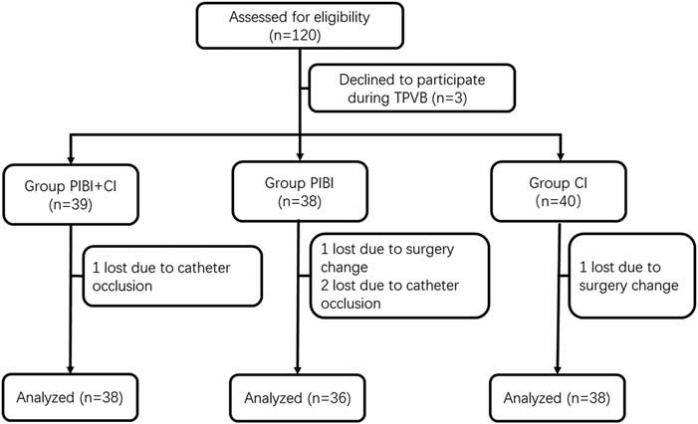
The CONSORT flow diagram. CI indicates continuous infusion; CONSORT, Consolidated Standards of Reporting Trials; PIBI, programmed intermittent bolus infusion; TPVB, thoracic paravertebral block.

The method of TPVB and catheterization were according to Fujii et al’s^18^ research and were performed for all patients per the following steps: (1) a C35X probe transducer (8 to 13 Hz curved; Sonosite Edge II, Bothell, WA) was placed at the T4-T5 or T5-T6 space (depending on the port site required for thoracoscopy), 2 cm lateral to the posterior median line to identify the T4 (or T5) transverse process and paravertebral space between the costotransverse ligament and pleura (Fig. 1A); (2) 2% lidocaine was applicated to infiltrate the insertion site, Then, a 19 G open tip needle (Teleflex, PA) was punctured from the skin to the paravertebral space, following by a 20 G catheter (Teleflex) with 3 cm beyond the needle tip; (3) a single bolus of 15 mL 0.4% ropivacaine plus 5 mg dexamethasone was injected through the catheter. The inferior movement of the pleura was identified to confirm the location of the catheter (Fig. 1B); (4) an intradermal tunnel was created to ensure the fixation of the catheter (Figs. 1C, D); and (5) 15 minutes later, the sensory assessment was performed, and TPVB catheter placement was regarded successful when the patient felt less pain than the contralateral side.

### Anesthetic Care

Electrocardiographs, invasive arterial pressure, pulse oximetry, and Bispectral Index (BIS) were routinely monitored during the surgery. General anesthesia was induced by intravenous injections of midazolam 0.05 to 0.2 mg/kg, propofol 1.5 to 2.5 mg/kg, sufentanil 0.5 μg/kg, and cis-atracurium 0.1 mg/kg. Double lumen tracheal intubation was performed upon BIS index fell to 40.

Anesthesia was maintained by CI of propofol (6 to 8 mg/kg/h) and remifentanil (0.3 μg/kg/min), with muscle relaxation achieved by intermittent administration of cis-atracurium. Controlled mechanical ventilation was adjusted to maintain the end-expiratory carbon dioxide of 35 to 45 mm Hg, and anesthesia depth was maintained at 40 to 50 BIS values. When the surgery began, the location of the catheter was reconfirmed by thoracoscopy to exclude any pleural injury or leakage of local anesthetics, which might impede the effectiveness of the TPVB (Fig. 1E). The infusion of all anesthetic drugs was discontinued upon skin closure.

### Intervention and Other Postoperative Analgesia

Previous studies have identified that ropivacaine is a safer choice for TPVB since it does not have any accumulation effect.^14^,^19^,^20^ In the present study, each of paravertebral block catheter was connected to a PCA pump (ZZB-1; AiPeng Medical Technology, Jiangsu, China) after surgery. the pump contained 300 mL of 0.2% ropivacaine, and the concentration and infusion rate of ropivacaine for the PCA pump were based on the Chen et al’s^14^ study. Detail, a researcher who was not involved in the anesthesia care set the parameters of the pump. For patients assigning to the PIBI+CI group, an intermittent bolus of 4 mL 0.2% ropivacaine was injected within 1 minute every hour followed with a CI at 4 mL/h; for patients assigning to the PIBI group, an intermittent bolus of 8 mL 0.2% ropivacaine within 1 minute was injected every hour; and for patients assigning to the CI group, a CI of 0.2% ropivacaine was performed at 8 mL/h. Besides that, the patient-controlled bolus dose was set at 5 mL in 1 minute with a lockout interval of 30 min for all groups. Additional 200 mL of 0.2% ropivacaine would be refilled if the volume of the pump had run out.

All patients were transferred to a thoracic surgical intensive care unit (TICU) after the surgery. If the patient recovered from the anesthesia with a Numerical Rating Scale (NRS)>4 (indicating moderate or heavy pain), while the PCA bolus dose failed to relieve the pain, an extra 5 mg of dezocine would be administrated intravenously for rescue.

### Outcomes

The primary outcome was the patient’s NRS score at postoperative 24 hours (T3), which appeared to be the most painful time point. The NRS score was assessed by asking patients to rank their pain severity from 0 to 10 (0=totally pain relief, 10=the worst pain ever) both at rest and coughing times. The secondary outcomes included the NRS scores at other time points (1 h after extubation [T1], postoperative 12 h [T2], 36 h [T4], and 48 h [T5]), the cumulative consumption of postoperative ropivacaine in the PCA pump within the following 2 days, the number of blocked dermatomes at postoperative 24 hours, and the number of patients who required extra dezocine for pain relief. The assessment of blocked dermatomes was performed by pinprick test using Von-Frey filaments with the comparison to the contralateral side. Exploratory outcomes included postoperative chest tube drainage time, length of TICU stay, and length of hospital stay. An investigator (X.H.) who was blinded to the randomization group assessed all outcomes.

### Sample Size

The required minimum sample size for this study was determined according to our preexperimental investigation and a previous study.^14^ Assuming the type I error (0.05) and type II error (0.1) with the power of the test of 90%, the number of patients needed in each group was 35. Hence, 40 patients were enrolled in each group to account for any unforeseen difficulties.

### Statistical Methods

Continuous variables with normal distribution were presented as mean±SD, while non-normally distributed continuous variables were presented as the median and interquartile range. The categorical variables were presented as numbers and percentages. For NRS scores, postoperative ropivacaine consumption, and blocked dermatomes, the Kruskal-Wallis test was used to analyze the differences among the 3 groups, followed by the Dunn test with Bonferroni correction to compare the difference between each pair.^21^ For the number of Dezocine rescues, the χ^2^ test was used to analyze the differences among the 3 groups and between each pair.

For exploratory outcomes, 1-way analysis of variance was used to analyze the differences among the 3 groups, followed by the Tukey test to compare the difference between each pair.

All analyses were performed using SPSS (version 19.0; IBM, NY), Prism (version 8.0.1; GraphPad Inc., CA), and R (version 3.5.3; R Foundation for Statistical Computing, Vienna, Austria) software. A *P*-value <0.05 was considered statistically significant.

## RESULTS

### Participant Recruitment and Patient Characteristics

From September 1, 2019, to December 30, 2019, 120 patients were recruited. Eight patients were excluded from the final analysis for the following reasons: consent withdrew in 3 patients; surgical type changed upon rapid pathologic results in 2 patients; and paravertebral block catheter occluded in 3 patients. Therefore, a total of 112 patients were analyzed (Fig. 2). The patient demographics and surgical characteristics are presented in Table 1, which were all comparable among the 3 groups. In general, the mean age of the included patients was 55 years old, with 63% (70/112) male patients.

**TABLE 1:** Patient Demographics and Surgical Characteristics

	PIBI+CI Group (N=38)	PIBI Group (N=36)	CI Group (N=38)
Age (y)	56±8	54±7	54±13
Sex (male/female)	23/15	26/10	21/17
Body mass index (kg/m^2^)	22.9±2.7	24.3±3.6	23.9±3.6
Duration of surgery (min)	154±50	159±57	167±72
Intraoperative sufentanil (μg)	38.5±9.0	39.5±8.1	41.2±9.0
Intraoperative remifentanil (μg)	2778±899	2866±1029	3009±1301

Data are presented as mean±SD.

CI indicates continuous infusion; PIBI, programmed intermittent bolus infusion.

### Primary Outcome

**FIGURE 3:**
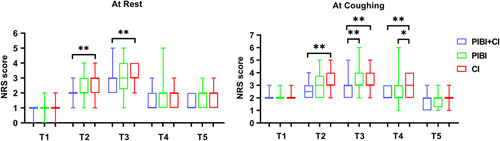
NRS scores among the 3 groups at each specific time point. *P<0.05; **P<0.01. CI indicates continuous infusion; NRS, Numerical Rating Scale; PIBI, programmed intermittent bolus infusion.

The median NRS scores at postoperative 24 hours (T3) were 3 in all groups (Table 2, Fig. 3). Nevertheless, there were significant differences regarding NRS scores among the 3 groups (*P*=0.009 and *P*<0.001 both at rest and coughing times; Table 2). Detail, the NRS score in the PIBI+CI group, was prominently lower than the CI group both at rest and coughing times (*P*<0.01; Fig. 3) and was also lower than the PIBI group during coughing (*P*<0.01; Fig. 3).

**TABLE 2:** Primary and Secondary Outcomes

	PIBI+CI Group (N=38)	PIBI Group (N=36)	CI Group (N=38)	*P*
Primary outcome (NRS score at postoperative 24 h)				
At rest	3 (2-3)	3 (2.8-4)	3 (3-4)**	0.009
At coughing	3 (2-3)	3 (3-4)**	3 (3-4)**	<0.001
Secondary outcomes				
NRS score at postoperative 1 h				
At rest	1 (1-1)	1 (1-1)	1 (1-1)	0.139
At coughing	2 (2-2)	2 (2-2)	2 (2-2)	0.162
NRS score at postoperative 12 h				
At rest	2 (2-2)	2 (2-3)	3 (2-3)**	0.002
At coughing	2.5 (2-3)	3 (2-3.3)	3 (3-4)**	<0.001
NRS score at postoperative 36 h				
At rest	1 (1-2)	2 (1-2)	2 (1-2)	0.054
At coughing	2 (2-3)	2 (2-3)***	3 (2-4)**	<0.001
NRS score at postoperative 48 h				
At rest	1 (1-2)	2 (1-2)	2 (1-2)	0.068
At coughing	2 (1-2)	2 (1.8-2)	2 (2-2)	0.256
Postoperative ropivacaine consumption (mg)				
Day 1	384 (384-384)	404 (384-424)**	404 (348-424)**	<0.001
Day 2	384 (384-384)	384 (384-384)	384 (384-404)*	0.018
Total	768 (768-768)	788 (768-808)**	798 (788-808)**	<0.001
Blocked dermatomes at postoperative 24 h	4 (4-4.8)****	4.5 (4-5)****	4 (3-4)	<0.001
Dezocine rescue	2 (5.3)	7 (19.4)	6 (15.8)	0.175

Data were presented as median (interquartile range) or n (%).

* *P*<0.05 compared with PIBI+CI group.

** *P*<0.01 compared with PIBI+CI group.

*** *P*<0.05 compared with CI group.

**** *P*<0.01 compared with CI group.

CI indicates continuous infusion; NRS, Numerical Rating Scale; PIBI, programmed intermittent bolus infusion.

### Secondary Outcomes

For the postoperative pain at other time points, patients in the PIBI+CI group did not show statistical differences in NRS scores at 1 hour after extubation (T1) and postoperative 48 hours (T5) compared with the PIBI and CI groups. In contrast, patients in the PIBI+CI group had significantly lower NRS scores than the CI group at postoperative 12 hours (T2) (*P*<0.01, both at rest and during coughing times) and 36 hours (T4) (*P*<0.01, at coughing time), and also had lower NRS scores than the PIBI group at postoperative 36 hours (T4) during coughing (Table 2, Fig. 3).

**FIGURE 4:**
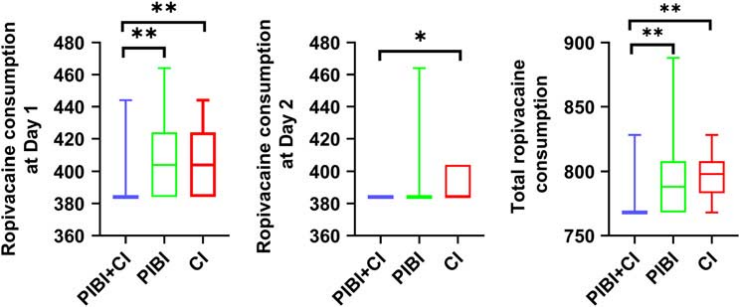
Cumulative dosage of ropivacaine consumption in each group. *P<0.05; **P<0.01. CI indicates continuous infusion; PIBI, programmed intermittent bolus infusion.

The medians of total postoperative ropivacaine consumption in the PIBI+CI, PIBI, and CI groups were 768, 788, and 798 mg, respectively, and the ropivacaine consumption among the 3 groups showed statistically different (*P*<0.01; Table 2, Fig. 4). Detail, patients in the PIBI+CI group, had lower ropivacaine consumption than both PIBI and CI groups at day 1 (*P*<0.01; Fig. 4), the CI group at day 2 (*P*=0.025; Fig. 4), and both PIBI and CI groups totally in 2 days (*P*<0.01; Fig. 4).

The numbers of blocked dermatomes in the PIBI+CI and PIBI groups were statistically comparable (*P*=0.28) and were both wider than the CI group (*P*<0.01; Table 2). In addition, a smaller proportion (not statistically significant) of patients in the PIBI+CI group (5.26%, 2/38) had required dezocine for pain relief when compared with the PIBI group (19.44%, 7/36) and the CI group (15.79%, 6/38).

For the exploratory outcomes, no significant differences were found in the chest tube drainage time (*P*=0.442), TICU stay (*P*=0.975), and hospital stay (*P*=0.370) among the 3 groups (Table 3).

**TABLE 3:** Exploratory Outcomes

	PIBI+CI Group (N=38)	PIBI Group (N=36)	CI Group (N=38)	*P*
Chest tube drainage time (d)	3.64±1.76	3.70±1.73	4.16±2.27	0.442
TICU stay (d)	1.09±0.82	1.12±0.54	1.11±0.81	0.975
Hospital stay (d)	10.61±4.09	10.94±3.13	9.79±3.53	0.370

Data are presented as mean±SD.

CI indicates continuous infusion; PIBI, programmed intermittent bolus infusion; TICU, thoracic surgical intensive care unit.

## DISCUSSION

### Interpretation

In this prospective randomized trial, we investigated the efficacy of the combination of PIBI and CI for TPVB on pain relief after thoracoscopic surgery. The results showed that the PIBI+CI approach reduced patients’ NRS scores from postoperative 12 to 36 hours, compared with either PIBI or CI approach alone. It should be also noted that although the extend of NRS score reduction was small (<1 point), the proportion of patients with NRS score ≥4 (considered to be moderate pain and needed additional medical intervention) in the PIBI+CI group was statistically less than those in the PIBI or CI group (shown in the Supplemental Table 1, Supplemental Digital Content 1, http://links.lww.com/CJP/A875). For secondary outcomes, patients in the PIBI+CI group had lower total ropivacaine consumption than those in the PIBI and CI groups. The width of the dermatomal sensory block in the PIBI+CI group was comparable to the PIBI group but wider than the CI group.

The study shed light on the optimal approach in pain management following thoracoscopic surgery. The results of this study suggest: (1) the combination of PIBI and CI provided a satisfactory pain relief after thoracoscopic surgery, which was slightly better than PIBI or CI alone; (2) the combination of PIBI and CI could reduce the postoperative anesthetic consumption, compared with PIBI or CI approach alone; (3) the combination of PIBI and CI could provide a comparable width of the dermatomal sensory block than PIBI, but wider than CI. Overall the results suggest that the combination of PIBI and CI for TPVB might be superior to both PIBI or CI alone.

There are a number of strengths of this study. We carefully considered the factors that may affect the results of the TPVB. First, we used the ultrasound technique during the needle placement, which is more reliable than the anatomic landmark^22^ or nerve stimulator technique.^23^ In addition, we reconfirmed the location of each catheter through the view of thoracoscopy in case of any pleural injury or leakage of local anesthetics. The success rate of TPVB catheterization in our study was 98.25% (112/114, 2 failures due to catheter occlusion), which indicated that the postoperative pain was unlikely to be attributed to the failure of the paravertebral block. Second, the same experienced anesthesiologist performed the TPVB in our study, which reduced the heterogeneity of clinical intervention. Third, we only enrolled patients undergoing elective unilateral thoracoscopic wedge resection with a single incision. It reduced the bias due to different extents of surgical trauma. Fourth, a previous study^18^ has demonstrated that it was difficult to insert the catheter beyond the ribs to the neighboring paravertebral space. Thus, the intercostal space we chose to block was in accordance with the location of surgical port decided by the surgeon based on the tumor site so as to gain the better analgesia effect. Finally, to determine settings for PCA, we referred to the previous researches in which the infusion rate was recommended ranging from 0.1 to 0.2 mL/kg/h and chose 0.15 mL/kg/h.^24^,^25^ In addition, we used 0.2% ropivacaine in this study, which has proven to be safe and effective for TPVB with no accumulative effect suggested by previous studies.^14^,^20^,^21^ Therefore, the results suggested by our study appear to be reliable.

### Generalizability

Thoracic surgery is painful. Without adequate pain control during the perioperative period, patients are reported to experience moderate to severe pain during the next 2 to 6 hours postoperatively.^6^ Alternatively, ultrasound-guided TPVB now has been recognized as a safe and reliable approach to treat postthoracotomy pain. The benefit of this technique includes improved analgesia, sparing opioids consumptions and the following side effects including vomiting, respiratory depression, urine retention, etc.^9^,^10^ A growing number of researches have proved that TPVB, either a single shot or continuous catheterization, could significantly alleviate the acute pain severity and prevent the risk of developing chronic pain thereafter.^26^,^27^ Compared with a single shot of TPVB that provides satisfactory analgesia only in the early postoperative time period (about the following 6 to 12 h according to previous research^28^ and our own experiences), continuous TPVB with catheterization provided prolonged, superior and patient-controllable analgesia. Moreover, we found in the present study that the most painful time for patients was 24 hours after the surgery, which might be beyond the effective analgesia duration of a single shot of TPVB. Thus, continuous TPVB might be a necessary and promising strategy to treat the pain after thoracic surgery.

In recent decades, several randomized trials have been performed to compare the efficacy between PIBI and CI approach for continuous TPVB. However, the results remained debatable. In this instance, the study by Chen et al^14^ demonstrated that PIBI with 0.2% ropivacaine at 8 mL/h (infused in 80 s) was superior to CI at 8 mL/h in providing a significantly lower NRS scale in the first 2 days, local anesthetics consumptions and greater patient satisfaction after thoracoscopic surgery. In contrast, Hida and colleagues^12^,^13^ found that PIBI with 0.2% levobupivacaine at 10 or 15 mL/3 h only provided a wider dermatomal blockage than CI at 5 mL/h, the NRS scales and opioids consumptions between these 2 approaches showed statistically comparable, indicating PIBI might provide equivalent pain relief to CI. To the opposite, if the infusion interval is prolonged to 5 hours or more, the superiority of PIBI to CI will diminish, even the reverse is observed.^19^,^29^ These findings suggest that types, concentration, infusion rate and intervals of local anesthetics might be crucial determinants for the efficacy of PIBI and CI.

There are several factors that may account for the differences in the efficacy between PIBI and CI. On the one hand, in the PIBI approach, the local anesthetics is infused rapidly to the paravertebral space at the early stage of the infusion interval by a higher infusion speed and pressure, which is expected to overcome the distance between the catheter tip and nerve distribution, thereby providing shorter onset time of analgesia and wider blocked dermatomes.^29^ However, a higher infusion rate may lead to increased absorption into the bloodstream, as demonstrated in the study by Hida and colleagues,^12^,^13^ in which the plasma concentrations of local anesthetics were rapidly increased in patients receiving PIBI infusion than CI. Moreover, catheterization would inevitably cause tissue damage at the paravertebral space as evidenced by the punctate bleeding that could be seen via thoracoscopy (Fig. 1E), which might accelerate local anesthetics absorption further. Hence, without any additional supplement, the concentration of local anesthetics at the paravertebral space might decrease rapidly at the later stage of the infusion interval, resulting in a reduced analgesic effect of PIBI. On the other hand, the CI technique involves a relatively lower infusion speed and pressure than PIBI, which therefore may not be as effective at the early stage of the infusion, but it ensures a constant infusion of local anesthetics that seemingly promotes equilibrium between local concentration and systemic absorption of local anesthetics. Therefore, we assumed that the combination of PIBI and CI might acquire the advantages of both techniques. In the present study, we found patients in the PIBI+CI group showed lower NRS scores and ropivacaine consumptions than either PIBI or CI group. It was intriguing because in the PIBI+CI group, the bolus of 4 mL 0.2% ropivacaine injected within 1 minute every hour seemed not to get the same effect with the bolus of 8 mL 0.2% ropivacaine injected within 1 minute in PIBI group, and the speed of CI in PIBI+CI group is also slower than that of CI group. We assumed it might be due to the following reasons. First, in our study, all procedures were conducted by a single experienced anesthesiologist as well as a surgical team, and only one thoracoscopic port for wedge resection was performed in all cases, which would have controlled and minimized levels of surgical trauma and severity of postoperative pain across all participants, therefore sparing the need of the dermatomal blocking range and making 4 mL 0.2% ropivacaine within 1 minute sufficient for the block. Second, the combined CI approach, like we discuss above, might provide an additional supplement of ropivacaine at the later stage of infusion intervals, which may strengthen the efficacy of PIBI. Nevertheless, we must conclude that the optimal delivery regimen for paravertebral infusion still remains unclear, thus the best working concentrations, infusion rates, and the ratio of PIBI/CI needs to be further elucidated.

In addition, the concentration, infusion rate and intervals of ropivacaine we chose in the present study for PIBI and CI were based on the study protocol by Chen et al.^14^ However, we did not observe the superiority of the PIBI to CI on pain relief as in the study by Chen and colleagues but discovered that the NRS scores of both the PIBI and CI groups remained comparable at 2 days following the surgery (except for postoperative 36 h during coughing time). The discrepancy could be attributed to the single bolus injection of local anesthetics during the operation. Contrary to the study by Chen et al^14^ (15 mL 0.375% ropivacaine was administered at 30 min before the completion of the surgery), we administered a 15 mL 0.4% ropivacaine plus 5 mg dexamethasone before skin incision. Previous studies have shown that patients who receive a single dose of the paravertebral block before skin incision have superior pain relief,^30^ in addition to steroids that have a promising additive effect in prolonging the therapeutic effect of local anesthetics.^31^ Therefore, we attributed higher concentration of ropivacaine combined with steroids injected before the surgical injury to better pain relief both in the PIBI and CI group in our study.

### Limitations

This present study had several limitations. First, only patients undergoing minimally invasive wedge resection were enrolled in this study. These patients generally had lower pain severity and therefore might not reveal subtle differences in the pain, Second, we did not list the incidence of complications, especially the toxic reaction of local anesthetics application because the concentration (0.2%) and the infusion rate (8 mL/h) of ropivacaine we used in the present study were lower than previous reports.^14^,^19^,^20^ Moreover all enrolled patients were going through general anesthesia, which may cover up the clinical manifestation of local anesthetics toxic reactions. Therefore, future studies should be stratified based on the surgery type and focus on testing the serial plasma concentrations of local anesthetics.

## CONCLUSIONS

The combination of PIBI and CI provided satisfactory and safe analgesia, which might be superior to PIBI or CI alone. Therefore, the combined use of PIBI and CI for the TPVB can be advocated for patients undergoing thoracoscopic surgery. Future study should focus on the concentration of blood local anesthetics and efficacy of PIBI+CI in different type of thoracic surgery.

## Supplementary Material

SUPPLEMENTARY MATERIAL

Supplemental Digital Content is available for this article. Direct URL citations appear in the printed text and are provided in the HTML and PDF versions of this article on the journal's website, www.clinicalpain.com.
