# Supplementary material for: Combined Programmed Intermittent Bolus Infusion With Continuous Infusion for the Thoracic Paravertebral Block in Patients Undergoing Thoracoscopic Surgery: A Prospective, Randomized, and Double-blinded Study
Source: Clin J Pain. 2022 Apr 20;38(6):410–7. doi: 10.1097/AJP.0000000000001037 (PMC9076251; doi:10.1097/AJP.0000000000001037)
Supplement: SUPPLEMENTARY MATERIAL [file ajp-38-410-s001.docx]

**Supplemental Table 1.** The percentage of patients with NRS≥4 in each group.

|  | PIBI + CI group  (n = 38) | PIBI group  (n = 36) | CI group  (n = 38) | P value |
| --- | --- | --- | --- | --- |
| NRS score ≥4 at 1-hour after extubation (T1) | | | | |
| At rest | 0 (0%) | 0 (0%) | 0 (0%) | 0.965 |
| At coughing | 0 (0%) | 0 (0%) | 0 (0%) | 0.965 |
| NRS score ≥4 at postoperative 12-hour (T2) | | | | |
| At rest | 0 (0%) | 5 (14%) | 2 (5%) | 0.045 |
| At coughing | 3 (8%) | 9 (25%) | 16 (42%) ^**^ | 0.003 |
| NRS score ≥4 at postoperative 24-hour (T3) | | | | |
| At rest | 3 (8%) | 11 (31%) ^*^ | 15 (39%) ^**^ | 0.005 |
| At coughing | 3 (8%) | 15 (42%) ^**^ | 18 (47%) ^**^ | <0.001 |
| NRS score ≥4 at postoperative 36-hour (T4) | | | | |
| At rest | 0 (0%) | 1 (3%) | 0 (0%) | 0.345 |
| At coughing | 0 (0%) | 5 (14%) | 12 (32%) ^**^ | <0.001 |
| NRS score ≥4 at postoperative 48-hour (T5) | | | | |
| At rest | 0 (0%) | 0 (0%) | 0 (0%) | 0.965 |
| At coughing | 0 (0%) | 0 (0%) | 0 (0%) | 0.965 |

Data were presented as number (percentage). PIBI, programmed intermittent bolus infusion; CI, continuous infusion; NRS, numerical rating scale.

^*^ P<0.05 compared to PIBI+CI group

^**^ P<0.01 compared to PIBI+CI group
